# LessStress - how to reduce stress in school: evaluation of a universal stress prevention in schools: study protocol of a cluster-randomised controlled trial

**DOI:** 10.1186/s13063-022-06970-x

**Published:** 2023-01-19

**Authors:** Christin Scheiner, Andrea Daunke, Alexandra Seidel, Sabrina Mittermeier, Marcel Romanos, Michael Kölch, Arne Buerger

**Affiliations:** 1 Department of Child and Adolescent Psychiatry, Psychosomatics and Psychotherapy, University Hospital of Wuerzburg, Würzburg, Germany; 2 German Centre of Prevention Research in Mental Health, University of Wuerzburg, Würzburg, Germany; 3 Department of Child and Adolescent Psychiatry, Neurology, Psychosomatics and Psychotherapy, University Medical Center Rostock, Rostock, Germany

**Keywords:** Stress, Stress reduction, Prevention, RCT, School, Resilience, Emotion regulation, Mindfulness, Self-compassion

## Abstract

**Background:**

Chronic stress is detrimental to health, and children and young people have had to cope with significantly more stress since the start of the COVID-19 pandemic. In particular, stress at school and in relation to learning is a major problem in this age group. Studies in Germany have indicated that the pandemic has led to a reduced quality of life (QoL) and an increased risk for psychiatric disorders in children and adolescents. Schools are an ideal setting for interventions against stress, which is one of the strongest predictors for the development of psychosocial problems. The present study seeks to address stress by means of a short prevention training programme in schools, including emotion regulation, mindfulness, and self-compassion. In addition to information material for self-study, students should have the opportunity to actively deal with the topic of stress and develop coping strategies within a short space of time. In contrast to very long stress reduction programmes that often last several weeks, the programme is delivered in just 90 min.

**Methods:**

The effectiveness of the short and economical prevention programme *LessStress* will be examined in a cluster-randomised controlled trial (RCT) encompassing 1894 students. At several measurement time points, students from two groups (intervention and control) will be asked about their subjectively perceived stress levels, among other aspects. Due to the clustered nature of the data, mainly multilevel analyses will be performed.

**Discussion:**

In Germany, there are no nationwide universal prevention programmes for students against stress in schools, and this gap has become more evident since the outbreak of the pandemic. Universal stress prevention in schools may be a starting point to promote resilience. By dealing with stress in a healthy way, mental health can be strengthened and maintained. Moreover, to reach at-risk students at an early stage, we advocate for a stronger networking between child psychiatry and schools.

**Trial registration:**

German Clinical Trials Register (DRKS) DRKS00025721. Registered on November 4, 2021

## Administrative information

Note: the numbers in curly brackets in this protocol refer to SPIRIT checklist item numbers. The order of the items has been modified to group similar items (see http://www.equator-network.org/reporting-guidelines/spirit-2013-statement-defining-standard-protocol-items-for-clinical-trials/).

**Table:** 

Title {1}	**LessStress – how to reduce stress in school** **Evaluation of a universal stress prevention in schools: Study protocol of a cluster-randomized controlled trial**
Trial registration {2a and 2b}.	German Clinical Trials Register (DRKS): DRKS00025721**,** registration date November 4^th^, 2021. https://www.drks.de/drks_web/navigate.do?navigationId=trial.HTML&TRIAL_ID=DRKS00025721 https://trialsearch.who.int/Trial2.aspx?TrialID=DRKS00025721
Protocol version {3}	Issue 1, date: 04^th^ November 2021 Protocol amendment number: 01
Funding {4}	The universal prevention programme is funded by the Kaufmännische Krankenkasse health insurer. The funder has no influence the collection, analysis or interpretation of data or the writing of this or any following article on *LessStress*. Contact address: KKH Referat Prävention und Selbsthilfe Herr Tobias Bansen Karl-Wiechert-Allee 61 30625 Hannover Deutschland
Author details {5a}	Christin Scheiner^1,2^, Andrea Daunke^3^, Alexandra Seidel^1,2^, Sabrina Mittermeier^1,2^, Marcel Romanos^1,2^, Michael Kölch^3^, Arne Buerger^1,2^ ^1^University Hospital of Wuerzburg, Center of Mental Health, Department of Child and Adolescent Psychiatry, Psychosomatics and Psychotherapy, Wuerzburg, Germany ^2^ German Centre of Prevention Research in Mental Health, University of Wuerzburg, Germany ^3^ Department of Child and Adolescent Psychiatry, Neurology, Psychosomatics and Psychotherapy, University Medical Center Rostock, Rostock, Germany
Name and contact information for the trial sponsor {5b}	Deutsches Zentrum für Präventionsforschung Psychische Gesundheit Julius-Maximilians-Universität Würzburg c/o Zentrum für Psychische Gesundheit Klinik für Kinder- und Jugendpsychiatrie, Psychosomatik und Psychotherapie Margarete-Höppel-Platz 1 97080 Würzburg Telephone: +49 931 201 78000 E-Mail: DZPP@ukw.de
Role of sponsor {5c}	The sponsor or the funding source had no role in the design of this study and will not have any role during its execution, analyses, interpretation of the data, or decision to submit results.

## Introduction

### Background and rationale {6a}

#### Background

The negative impact of the SARS-Cov2 pandemic on adolescents’ mental health and QoL is becoming increasingly apparent. Recent studies in Germany and Austria have revealed an alarming decrease in QoL and an increase in the risk of developing psychiatric disorders among children and adolescents [1, 2]. School is the natural environment for cognitive and social learning in young people, but several lockdowns and school closures over the past pandemic school years have exerted a strong impact on students’ learning: Learning goals have generally not been reached, and students from disadvantaged backgrounds have been especially impacted [3, 4]. The digitalisation of education had been rather hesitant prior to the pandemic, and it remains an enormous challenge for schools, teachers, students, and their parents [5]. It is clear that the past 2 years have led to increased stress for young people, which must be surmounted given the students’ increasing knowledge gaps.

One extremely important conclusion that can be drawn from the ongoing pandemic situation is that children and young people are suffering [6–8] due, among other factors, to adverse home conditions, family stress, or mental illness. Therefore, it is essential to provide rapid assistance and to develop and evaluate effective, efficient prevention programmes that are easy to access and implement [6]. As child and adolescent mental health is hugely multifaceted [7], we limit ourselves here to one risk factor of which we are all aware, and with which students have struggled a great deal over the last 2 years: stress [1, 2]. A very broad definition of stress goes back to Hans Selye, a twentieth-century physician, biochemist, and hormone researcher, who said “Stress is the nonspecific response of the body to any demand” [9]. There are many different definitions of stress depending on the context. In the behavioural sciences, stress describes the perception of a threat, which is reacted to with anxiety, emotional tension, and discomfort. Neuroendocrinological stress can be described as a reaction of the body, more precisely the sympathetic nervous system, to release ACTH and adrenal glucocorticoids in response to a stimulus [10]. In the psychological context, it is assumed that the induction of negative affective states (e.g. feelings of anxiety and depression) influences the pathogenesis of physical illnesses [11]. Chronic stress is likely to result in long-term or lasting changes in emotional, physiological, and behavioural responses, which in turn affect susceptibility to and progression of disease, including stressful events such as trauma [12]. The endocrine response triggered by stress is composed of two endocrine response systems that are particularly responsive to psychological stress: the hypothalamic-pituitary-adrenal (HPA) axis and the sympathetic-adrenal medulla (SAM) system [13, 14]. Cortisol as the primary effector regulates a wide range of physiological processes. The repeated and prolonged activation of the HPA and SAM systems can severely impair physiology and is therefore at increased risk for physical and psychiatric disorders [11]. Symptoms associated with stress include loss of appetite, headache, stomach pain, difficulty concentrating, sleep disorders [15], and a decrease in QoL [16]. Stress is perceived differently from person to person, has a cumulative effect, and is seen as one of the strongest predictors of mental illness [15, 17]. A survey of around 230,000 6–18-year-olds conducted by the Kaufmännische Krankenkasse in 2017 [18] revealed a significant increase in mental disorders among children and adolescents with stress being identified as the major issue. Another prospective cohort study reported depression or neurotic and somatoform disorders to be the main disorders comorbid to stress [19]. This finding is supported by a body of evidence conducted in other studies [20–22]. The increased stress in young people pertains not only to school and academic achievement, but also to further factors such as stress with parents (regarding both school and home life), overstimulation by media, bullying in social networks, or general fears, which all contribute to a higher stress perception. School and school-related stress is certainly a major concern, even in elementary school: Young children are vulnerable to stress and show stress symptoms, and it has been identified that girls are more susceptible to stress and are coping differently compared to boys [20, 23]. Stress levels among children and adolescents appear to be higher in more academic school types than in vocational schools [24], and rise during adolescence [25]. With respect to school, and specifically stress caused in school, it is necessary to address and prevent anxiety (separation anxiety, fear of school, social anxiety, and test anxiety) together with the students themselves [26]. Crucially, moreover, stress leads to neurocognitive alterations [27].

But is stress prevention effective and feasible? In a recent study examining the mediating effects of mental and physical health problems on health care costs in a sample of German school (*N* = 284, *M* = 16.75 ± 0.64 years), Eppelmann and colleagues found that adolescents’ perceived stress had a significant effect on overall health care costs. An increase of stress by 1 *SD* increased the chances for costs by an odds ratio of 1.39. This effect was fully mediated by mental health (problems) [28]. Mental illnesses are the second largest cost drivers in the German health system [28, 29].

Prevention research has yielded mixed findings regarding school programmes targeting stress (stress management, coping skills, mindfulness/awareness, relaxation, life skills training): While meta-analyses have found some to be effective [30, 31], other meta-analyses have shown different results [32, 33]. However, due to methodological limitations within these reviews, more research is necessary. So far, the meta-analyses in this area have led to the following major conclusions:

- The tested programmes were quite long (up to 50 min per session over several weeks, 8–30 sessions)
- The frequency of sessions was very high (just 10 min per session but implemented several times a day, or up to 42 times)
- Sample sizes were very low (21 to 323)
- Follow-ups were often missing
- Measurement of stress symptoms was not incorporated
- Not aimed at adolescents of middle-school age
- Lack of internal consistency for the applied measures
- It may be necessary to integrate parents into stress prevention programmes

In sum, while children and adolescents did gain more knowledge about stress and coping with stress, the programmes did not reduce self-perceived stress. One approach is mindfulness at school, which has become highly popular in the USA and the UK, where there are even mindfulness-based apps for teachers and students [34]. A recent stress prevention programme for German students (3× 45 min) revealed significant effects in terms of knowledge gains but no reduction in self-perceived stress between the control and intervention groups, although the students did like the programme and would recommend it [35]. The authors stated that in contrast to their prevention programme, other interventions such as mindfulness-based stress reduction (MBSR) [36], when executed more than 20 times, or a programme lasting for 10 units [37], were effective in stress reduction. They concluded that in order to acquire individual stress coping models, students need more time to generalise to their own situation. At this point, the following question arises: How do the effectiveness, required effort, and practicability of a programme relate to one another? For example, what can we achieve with a combination of psychoeducation and group work? With *LessStress*, we take the approach of conveying knowledge about stress by means of a booklet and targeting stress-coping strategies through one training unit.

#### Reason for the trial

The COVID-19 pandemic has made the urgent need for stress prevention in adolescence even more apparent. In the field of prevention research, there have long been calls for more, and especially effective programmes for children and adolescents [38], given the frequent onset of mental disorders in adolescence, the persistence of the disorders, and a particular vulnerability in this age group [39, 40]. We are currently faced with a situation of many children and adolescents with high levels of stress, especially concerning school [18, 41]. Life during the pandemic has significantly exacerbated stressful situations at home and at school. Meta-analyses have demonstrated the effectiveness of stress prevention programmes at schools, but due to the acute nature of the situation, we need to evaluate short, easy-to-implement, and cost-effective programmes [38, 42–45]. *LessStress* differs from the programmes that have been developed and evaluated so far: It saves time and money, is easy to implement in schools, and combines different approaches such as skills-based methods, mindfulness, and preventive measures. In view of the high levels of self-perceived stress among children and adolescents, with the pandemic multiplying the challenges at school, effective prevention is essential.

## Objectives {7}

### Research hypothesis

The universal prevention program *LessStress* reduces students’ subjective stress experience and can thus help to maintain or strengthen their mental health.

### Objectives

In order to find out whether the universal prevention programme *LessStress* has a significant impact on students’ subjective experience of stress, the intervention group (IG) will be compared with a waiting list control group (WL) in terms of scores on the *Perceived Stress Scale 10* (PSS-10) [46, 47].

We expect that students who take part in the universal prevention programme *LessStress* will significantly benefit, as measured by the primary and secondary outcomes. Given the preventive nature of the programme, we expect to find a decrease in self-perceived stress and fewer mental health problems in the IG after 6 months, which will be assessed with the secondary outcomes.

To test the efficacy, we will collect data using self-administrated questionnaires, which will be implemented within an online survey. The questionnaires are standard instruments in the field of psychology and show good psychometrics properties. Questionnaires remain the means of choice to assess personal states and feelings.

#### Primary objectives

Self-perceived stress will be assessed using the German 10-item version of the *Perceived Stress Scale* (PSS-10) [47]. We expect a significant decrease for the primary outcome “self-perceived stress” in the IG.

#### Secondary objectives

In addition to the primary outcome, the evaluation of *LessStress* will include the following secondary outcomes: emotion regulation, self-compassion, sleep quality, depression index, anxiety index, self-injurious behaviour, eating disorder symptoms, and health-related quality of life. To investigate adolescents’ resources, we will assess protective and resilience factors (see Table 1 for further details).

**Table 1:** Overview of the questionnaires being used

**Instrument**	**Content**	**Length**
**PSS-10:** *Perceived Stress Scale* (primary outcome measure)	Primary objective Developed by Cohen, Kamarck, and Mermelstein, translated and validated by Klein, Brähler et al., 2016 [47]. Investigates the degree to which life was unpredictable, uncontrollable, and overwhelming within the last month. Very good internal consistency (*a* = .84). Positive correlation with depression and anxiety, negative one with quality of life.	10 items
**DERS-SF***: Difficulties in Emotion Regulation Scale Short Form*	Measures emotion (dys-)regulation multidimensional. According to the model by Gratz and Roemer [48], the subscales “non-acceptance”, “problems with target oriented behaviour”, “impulsive conduct problems”, “lack of emotional awareness”, “limited access to emotion regulation strategies”, and “lack of emotional clarity”.	16 items
**SCS:** *Self-Compassion Scale*	The Self-Compassion Scale was developed by Neff in 2003 with 26 items. The shortened version showed satisfactory to good psychometric properties so far [49].	12 items
**WHO-5***: WHO Fragebogen zum Wohlbefinden*	The facets of psychological well-being like mood, interest, energy, and drive within the last 2 weeks are assessed. Used worldwide, non-invasive questions, translated in 30 languages, the first version was published in 1998. Very good internal consistency with *α* = .85. German version by Allgaier [50].	5 items
**PHQ-4***: Gesundheitsfragebogen für Patienten – Depressionsmodul*	The PHQ-4 is a valid screening instrument for symptoms of depression and anxiety symptoms (Cronbach α >0.80) [51]. Responses are scored as 0 (“not at all”), 1 (“several days”), 2 (“more than half the days”), or 3 (“nearly every day”). PHQ–4 scores go from normal (0 –2) to mild (3–5) to moderate (6–8) to severe (9 –12). Symptoms within the last 2 weeks are being interrogated.	4 items
**DSHI-9***: Deliberate Self-Harm Inventory*	An initial question is asked whether someone has already intentionally injured his-/herself. If this is answered with yes, the type, onset, and frequency of self-harm are obtained [52].	1–6 items
**ISI***: Insomnia Severity Index*	Measures the quality of sleep and goes back to [53]. Difficulties falling asleep or staying asleep and the impairment they cause in everyday life are in the focus.	7 items
**SEED***: Short Evaluation of Eating Disorders*	It obtains the central symptoms of anorexia (underweight, fear of weight gain, impaired body awareness) and bulimia (binge eating, compensatory measures, excessive preoccupation with figure and weight) within the last month [54]. It has already been used in student samples with good experience.	6 items
**SFF***: Schutzfaktoren*	This short questionnaire captures three scales of protective factors that correlate significantly with data from the SDQ. Children who have more social, personal, and family resources are correspondingly less conspicuous in the SDQ [55].	12 items
**SISE:** *Single-Item Self-Esteem Scale*	This 1-item version goes back to the 10-item scale of the Rosenberg Self-Esteem Scale (RSES) developed by Rosenberg in 1965. The original sheet captured self-esteem with 5 positive and 5 negative questions. Recent studies have also demonstrated very good measurement properties for the 1-item version [56]. The question asks how well the statement “I have high self-esteem” applies to oneself. A 4-point Likert scale is then presented.	1 item
**CD-RISC-10:** *Connor-Davidson Resilience Scale-short form*	The long version was designed by Connor-Davidson in 2003 to measure resilience. Resilience means the ability to cope with internal or external stressors. The CD-RISC is widely used and has good psychometric properties (*a* = .84) [57]	10 items
**FAS III:** *Family Affluence Scale*	The FAS III measures the socioeconomic status using 6 questions and has very good quality criteria. Furthermore, the MacArthur scale question is connected, which asks about the subjective assessment of the prosperity of the adolescents’ own family [58]	6 items + 1

Finally, we will record demographic variables (e.g. age, gender) and socioeconomic status.

### Aims

The study will be carried out with 1894 adolescents at different schools in the Federal states of Bavaria, Mecklenburg Western Pomerania, and Hesse and encompasses the following aims:

- Improving stress managing skills in students
- Enhancing students’ quality of life and their mental health

## Trial design {8}

**Fig. 1:**
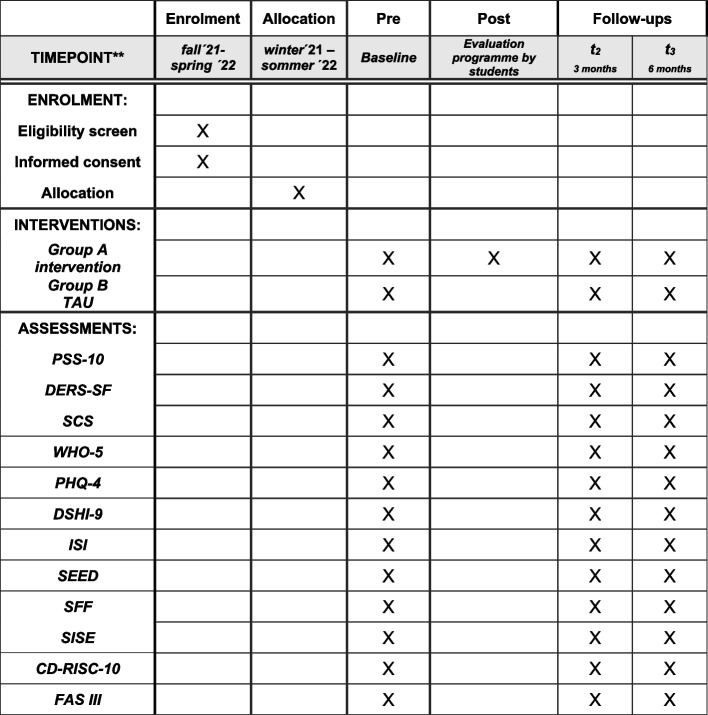
Timetable for the study *LessStress*

The *LessStress* trial is designed as a cluster-randomised, controlled, non-blinded superiority trial with two parallel groups and a primary endpoint of self-perceived stress after 6 months. We will perform a random assignment on the school level with a 1:1 allocation ratio. The study will last for 6 months and will include four measurement time points: Pre = baseline, Post = student and teacher evaluation, T2 = 3-month follow-up, and T3 = 6-month follow-up (Fig. 1).

### Randomisation

Randomisation will be performed at the school level in order to avoid any interclass interference within the intervention and waiting list control group. To reduce bias, we will be supported by a co-worker of the clinic (Hans Aster), who is not involved in this project and a member of another working group (Cognitive and computational Neuroscience), and will perform the randomisation using Microsoft Excel.

## Methods: participants, interventions, and outcomes

### Study setting {9}

The study will be conducted in the Federal states of Mecklenburg-West Pomerania, Bavaria, and Hesse [59]. Due to the broad geographical sample and the wide age range of 12–18 years, we will be able to obtain a well-generalisable epidemiological overview. The number of participating schools or classes cannot be predicted at this stage, for the following two reasons: (1) the pandemic has dramatically increased the stress and workload in schools, which is associated with less participation in non-school projects. (2) We are focusing on direct contact with teachers, school psychologists, and school social workers to bring the program to their schools, which can lead to more time required for recruitment.

### Eligibility criteria {10}

#### Inclusion and exclusion criteria

Participants must be aged between 12 and 18 years and be in the 6th to 12th grade. We chose this age group for two reasons: (1) The onset of adolescence brings about a clear increase in social, cognitive, and emotional challenges and expectations. Furthermore, neurological and cognitive change processes are underway [60]. Taken together, this can correspondingly increase self-perceived stress. (2) Epidemiological data show that the end of childhood, the beginning of adolescence, and adolescence itself represent the period with the highest risk of onset of mental disorders [28, 61].

Only one exclusion criterion will be applied: lack of informed consent from the adolescents or their parents/guardians. Moreover, we will assess the presence of a psychiatric illness with accompanying psychotherapy in order to exclude these students from data analysis.

### Who will take informed consent? {26a}

For the trial, the informed consent of the respective schools’ head teachers is required, as well as the informed consent of a legal guardian and of the students. If schools express an interest in participating, a meeting will be organised in order to provide detailed information about the study. In the case of participation, the legal guardians of students in the participating grades/classes will be given detailed written information sheets and will be able to watch an online presentation, in which the study is orally explained. The students will receive posters with information. If students wish to participate, they will provide their written consent by clicking on “Yes, I do want to participate in the study” on the first page of the online survey.

### Additional consent provisions for collection and use of participant data and biological specimens {26b}

Not applicable. No biological specimens will be collected.

## Interventions

### Explanation for the choice of comparators {6b}

In prevention research, a comparison of an intervention group and a waiting list control group is common for ethical reasons [42]. In order to test the effectiveness of a universal prevention program, comparing these two groups seems reasonable and cost-effective. As we will be working with adolescents, we will ensure that every school in the waiting list control group will be offered the intervention directly after the final assessment (6-month follow-up), enabling all adolescents to eventually benefit from the programme.

### Intervention description {11a}

#### Intervention

*LessStress* - A universal prevention programme in school - was designed by a team of psychologists, scientists, and psychiatrists with a focus on preventing mental health problems in adolescents. The intervention consists of two parts: a 90-min interactive lesson in class, which is combined with a booklet, called *Stresserwizzer*. The 90-min lesson will be presented to the class by school staff, who will receive corresponding training in advance. The lesson is divided into three main topics of emotion regulation, mindfulness, and self-compassion. Students will be given background information (what are emotions, how do we know what we are feeling, how are our emotions linked to our body, thoughts and behaviour, how can we change emotions/what is mindfulness, how can I be mindful in my daily life/what is self-compassion, how can I be more compassionate) and exercises on each topic. All exercises can be done alone or in small groups. Each student receives a workbook that contains information conveyed in the lesson as well as the exercises themselves. An important topic discussed with students is their ability to use skills. The idea is to have the class discuss what they have been doing to maintain their health so far or what they usually do to improve their mood. In a next step, students should think about other things/strategies they could try out in the future. It is important to connect the things they have learned theoretically about controlling their own emotions with things they can apply and integrate into their daily lives. The open discussion in class should also be beneficial to students who have used fewer skills so far and are less integrated in terms of social life. By hearing others talk about their experiences, they might want to try new things. In a next step, mindfulness is discussed, and the students experience different types of meditation. They are asked to think of situations in their daily lives when they have already been mindful and to think of situations to be more mindful in. Following the topic of mindfulness, self-compassion and also the connection to the self-critical inner voice will be discussed (what is the difference between self-pity and self-compassion, do I know this critical inner voice in me, what does it do, how can I deal differently with defeats or mistakes in the future). The main basis of this intervention is dialectical behavioural therapy for adolescents (DBT-A [62–64]) as well as studies evaluating the effectiveness of mindfulness for stress reduction [30, 31]. The aim of this lesson is to (1) convey skills that can help students protect themselves from stress and its consequences using a rather open approach and (2) address the topic of stress in teenagers and stress in school. The “rather open approach” here refers to experiential education content: For the most part, the students need to work out the content for themselves and not be silent listeners (e.g. group work, small-group or the whole-class discussions, and so on). Actively involving the students in the process of the lesson should ultimately enable the content to be better linked and remembered. Particularly regarding the topics emotion regulation and self-compassion, we want the students to experience different strategies and find out what works best for them individually. For most students, it is very likely that *LessStress* will be the first time they have done a mindfulness exercise, as mindfulness is not yet very common in German schools. In addition, students are given a 40-page booklet that contains useful information about stress in general and specifically about stress at school, along with helpful advice about what to do when feeling overwhelmed [65, 66]. This booklet has a modern design targeted at arousing adolescents’ interest. Students should read the booklet by themselves, and teachers should refer to the booklet from time to time to bring it back to the students’ minds. The posters of the study, which serve to provide information for the students about the purpose and process of the study, also fulfil another task: Through their presence in the classroom, they provide a “daily reminder” and better transfer of the content from the lesson and booklet into students’ daily lives.

### Criteria for discontinuing or modifying allocated interventions {11b}

All participating schools will be supported by our study team during the implementation of the programme. Experience from other studies conducted in schools has shown that weekly contact by email and telephone is a good way to stay in touch and to clarify questions quickly. Through regular exchange and contact with the teachers carrying out the programme, difficulties should be detected and corrected in advance in order to improve adherence.

### Strategies to improve adherence to interventions {11c}

#### Adherence

Each participating school (and especially the staff) will receive online training before administering the programme. This training will focus on the implementation of the PowerPoint presentation guiding the lesson and will also refer to the booklet. Furthermore, two to three small role-plays on crises will be carried out to prepare the teachers for all eventualities, such as students reporting non-suicidal self-harm to the teachers or students becoming highly stressed (e.g. starting to cry, needing to leave the room). To check adherence during the intervention, we will ask the school staff to fill out an online survey measuring their adherence to the given instructions. Questions will be asked about the presentation itself, how well students have understood the topics, whether everything was discussed, what questions remained, how easy it was for the teachers, and how well they think the class liked it.

In a next step, this information should be used in order to improve the presentation if necessary.

### Relevant concomitant care permitted or prohibited during the trial {11d}

During the implementation phase of *LessStress*, no other prevention programme should be carried out in the participating classes at the schools during the relevant period. Normal teaching and the general curriculum are, of course, allowed.

### Provisions for post-trial care {30}

One positive side effect of this trial is the networking between schools and clinicians. We aim to reduce stigmatisation and encourage communication. If any student requires healthcare during or after the study, the Department of Child and Adolescent Psychiatry of Rostock will offer appointments. We are confident that by being present and talking to the schools and students, we can lower the threshold, enabling students to seek help more frequently and faster.

### Outcomes {12}

#### Primary outcome

- Differences between the two groups (IG and WL) regarding self-perceived stress measured with the *Perceived Stress Scale* (PSS-10) [46, 47, 67] at 3- and 6-month follow-ups.
- Scores on the PSS-10 range from 0 = *no stress at all* to 40 = *maximum stress*. We assume a significant reduction in the intervention group.

#### Secondary outcome

- Furthermore, we assume that adolescents, who show a lack of emotion regulation skills measured with the short form of the *Difficulties in Emotion Regulation Scale* (DERS-SF) [48], will show higher levels of stress, lower QoL, and more mental health problems. The DERS-SF yields a sum score out of 18 items ranging from 0 = *no difficulties at all* to 90 = *extreme lack of regulation strategies*.
- We assume a correlation between self-perceived stress and self-compassion insofar as the more stress students have, the less self-compassion they show. Therefore, we will measure self-compassion with the German short form of the *Self-Compassion Scale* (SCS-SF) [49].
- We assume that a lower subjective well-being (WHO-5) [68] will be associated with higher stress levels among adolescents.

The same patterns are expected for psychopathological measurements such as depressive and anxiety symptoms (PHQ-4) [51], eating disorder symptoms (SEED) [54], and poor sleep quality [53, 69]. In order to verify these hypotheses, we will look at the symptom level itself and will also try to create a “psychopathology-score” (PP score) for each participant. This score will be determined by grouping: For every questionnaire, a scale will be drawn up with three different ratings (normal, abnormal, and pathological) by splitting at the 25% and 75% confidence intervals, with normal = 0 points, abnormal = 1 point, and pathological = 2 points. This way an individual score can be calculated for each participant representing his or her psychopathological level of health/illness. Furthermore, we assume the lower the HrQoL, the higher will be the PP score.

### Participant timeline {13}

**Fig. 2:**
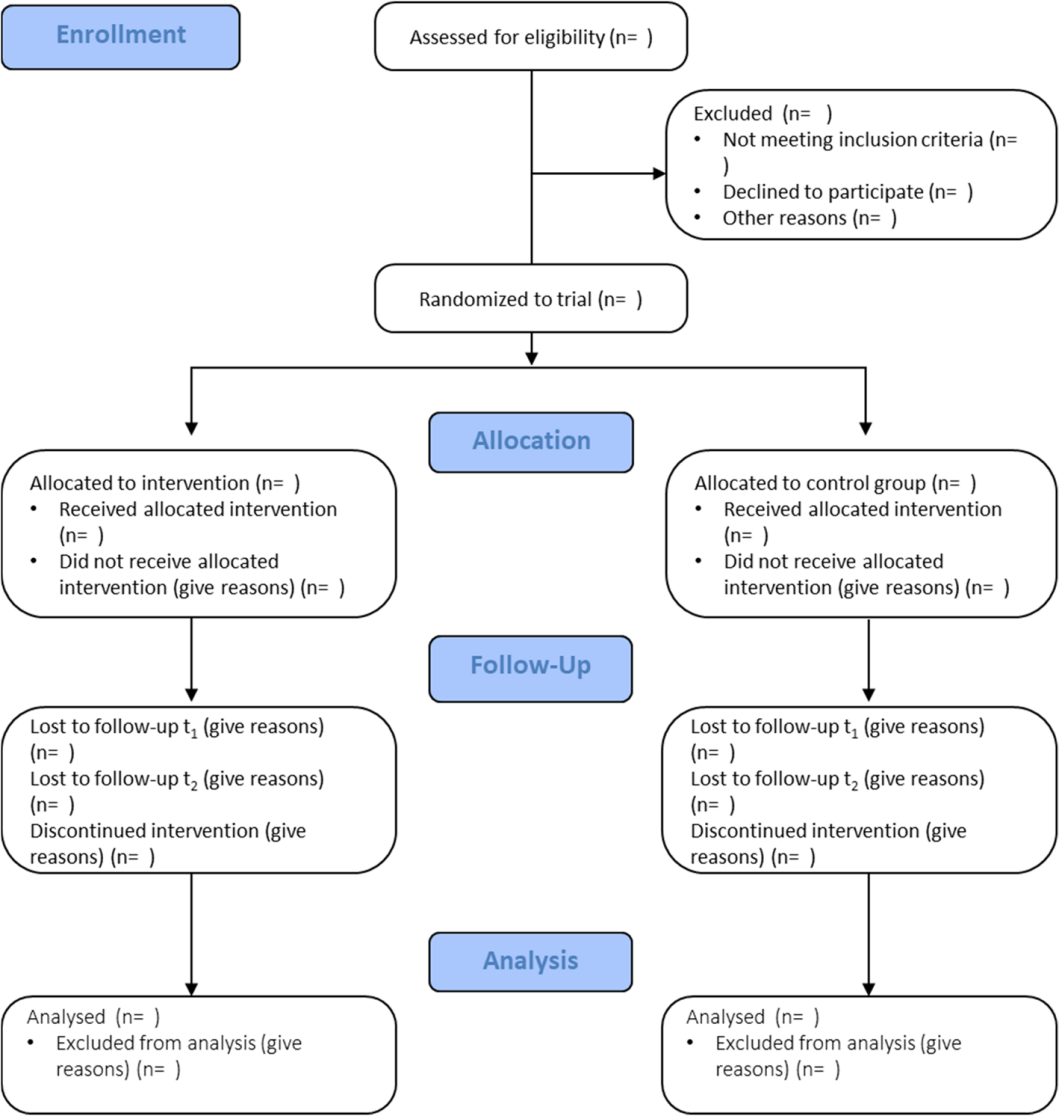
Study flow chart

All participants will be assessed using the full battery of instruments as displayed in Table 1. The data assessment will take place 14–30 days after randomisation. For the intervention group (IG), the universal prevention programme *LessStress* will be administered by school staff within the 21 days after baseline. Post-assessment is due within a week after participating the lesson of *LessStress*. Follow-up interviews, using the full battery of questionnaires, will be conducted at 3 months (T2, 104–120 days) and 6 months (T3, 194–210 days) after randomisation. A 14-day window (± 7 days) will be permitted within which to schedule the time points for T2 and 3. For a graphic overview of the study, please see the flowchart in Fig. 2.

### Sample size {14}

To analyse cluster-randomised trials, specific constraints must be followed, as individuals within a cluster are assumed to be more similar compared to individuals from different clusters. In order to detect significant effects, the sample size of the trial must be adjusted. This so-called clustering effect (1 + (*m*-1)×*p* [*m* = average number of subjects per cluster; *p* = intraclass correlation coefficient (ICC)]) resulted in an estimated *N* = 1795. For *m*, we assumed an average of 80 within an entire grade as the average class size in Germany varies nationwide (21.2–30.4 students per class), and the ICC was estimated quite conservatively at approximately 0.05 [70] on the basis of the primary outcome. Hence, a clustering effect of 4.95 was determined. Assuming a small effect size (Cohens *d* = 0.2) in universal prevention based on previous studies [71], with a significance level alpha = 0.05 and a power = 0.9, g*power suggests a total of *N* = 306 [72]. Considering the clustering effect, we have to multiply this by 4.95, reaching an overall *N* = 1515. Assuming a dropout rate of 25% due to moving out of the area, sickness, or other reasons, we must add another 379, leading to the final *N* = 1894.

### Recruitment {15}

The recruitment will take place in autumn 2022. Schools are the most appropriate setting for universal prevention for the following reasons: (1) An adolescent’s life is mostly played out at school, (2) compulsory school attendance provides the opportunity to reach a large number of adolescents, and (3) there is evidence that students are more likely to accept such programmes within school than during their free time. The School Boards will help us to get in touch with eligible schools. *LessStress* targets classes from the 6th to 12th grade at public and private schools. Any school principal or council expressing an interest can officially register for potential participation via mail. In a next step, further information material regarding the study will be sent out if required. If schools agree to participate, a letter of consent has to be signed, stating how many grades and classes will participate in *LessStress*.

## Assignment of interventions: allocation

### Sequence generation {16a}

Participating schools will be randomly assigned to one of the two groups using Microsoft Excel. The randomisation will be carried out on the school level following a 1:1 allocation ratio. This enables interference between the IG and WL to be avoided.

### Concealment mechanism {16b}

The schools will be immediately informed about the allocation via mail. For randomisation, we will use Microsoft Excel. To proceed with organising the appointments and the intervention, schools have to know to which group they have been allocated. The allocation will be executed in the presence of a third independent employee of the clinic (SM).

### Implementation {16c}

As there are no patients involved in this study, we do not expect a risk of bias associated with the methods of sequence generation and/or inadequate allocation concealment. Schools do have to know in which group they are in, in order to proceed with the organisation of appointments and intervention.

## Assignment of interventions: blinding

### Who will be blinded {17a}

Due to the study design and the planned intervention, there will be no blinding. Schools need to know the group to which they have been allocated in order to plan and organise the next steps within the study. We do not expect any ascertainment bias shown in the answers to the questionnaires between the two groups.

### Procedure for unblinding if needed {17b}

Not applicable. Due to the study design, schools and participants will know the group to which they have been assigned. However, to avoid manipulation of the questionnaires, participants will be unaware of the study hypotheses. The WL control group will merely be told that the intervention will take place in 6 months and that data will be collected in the interim.

## Data collection and management

### Plans for assessment and collection of outcomes {18a}

#### Data collection

The baseline data collection is scheduled for autumn 2022, if permission and recruitment are completed in time. The intervention should begin immediately after baseline data collection. During this time, the usual curriculum will be run in the waiting list control group. The post-data assessment will take place within 7 days post-intervention. The first follow-up will take place 3 months post-intervention (spring 2023) and the second follow-up will take place 6 months post-intervention (summer 2023). If permission and recruitment take longer than expected, e.g. due to possible pandemic-related restrictions, we plan to extend the recruitment phase until spring 2023.

#### Measures- questionnaire

Data collection will be conducted anonymously through a self-administered questionnaire completed on participants’ own mobile devices either in school or at home. If no mobile device is available at school, students will either receive a loan device or will be able to use the school’s IT room. Each student will generate an individual 12-item code consisting of numbers and letters based on different questions. This code can be used to link data from the three measurement time points without the need for a list combining name and data set, meaning that data security is guaranteed at all times. We have chosen to use mobile devices for the following reasons: (1) careful use of environmental resources, (2) greater willingness and enthusiasm to fill out the questionnaires on the part of the students, and (3) fewer missing data. A list of all questionnaires is presented in Table 1. Each questionnaire has been selected carefully (validated among the target group, sufficient to good psychometric properties, aimed at assessing a targeted construct, no iatrogenic effects known so far, use of an adapted version for adolescents whenever possible).

For the assessment of our primary outcome stress, we will use the 10-item version of the *Perceived Stress Scale* (PSS-10; original by Cohen, Kamarck, and Mermelstein [67], German validation by Klein, Brähler et al. [47]; *α* = .84). The PSS-10 examines the degree to which life seems unpredictable, uncontrollable, and overwhelming, with responses referring to the last month. PSS-10 has been found to correlate positively with depression and anxiety negatively ones to QoL.

Emotion regulation will be assessed using the *Difficulties in Emotion Regulation Scale* DERS-SF [48], an 18-item short version of the original 36-item version [73, 74]. The DERS-SF measures emotion regulation on six dimensions and has shown a Cronbach’s *α* of .90.

To assess self-compassion, we will use the total summed score of the short form of the *Self-Compassion Scale* (SCS-SF) [49]; the original *Self-Compassion Scale* (SCS) was developed by [75, 76]. The original English-language 12-item short form has shown adequate internal consistency (Cronbach’s *α* > .86) and a near-perfect correlation with the long-form SCS. Within a German sample and for the German validation, the SCS also showed good internal consistency for the subscales, ranging from .71 to .79 [76].

The *Insomnia Severity Index* [69] was developed by Morin [77] and the German validation was conducted by Gerber [53]. It is a useful screening instrument consisting of seven items, including night-time and daytime components of insomnia. A Cronbach’s *α* of .76 has been reported for boys and girls.

The four-item Patient Health Questionnaire (PHQ-4) is a brief screening scale for symptoms of depression and anxiety, showing good internal consistency (Cronbach's α > 0.80) [51, 78]. The PHQ-4 is a combination of the 2-item Patient Health Questionnaire (PHQ-2) [79, 80] and the 2-item Generalized Anxiety Disorder screening tool (GAD-2) [81]. Questions are answered on a scale ranging from 0 to 3 and result into mild (3-5), moderate (6-8), and severe PHQ-4 categories (9-12).

The *Short Evaluation of Eating Disorders* (SEED) [54] consists of six items exploring the main symptoms of anorexia nervosa (underweight, fear of gaining weight, body image disturbance) and bulimia nervosa (binge eating, compensatory behaviour, preoccupation with body shape and weight) within the last month or week. The SEED has previously been employed successfully in school students [82].

The five-item *World Health Organization Well-Being Index* (WHO-5) was first published in 1998 and has been translated into more than 30 languages. It measures subjective well-being and was derived from the WHO-10 [83], which was itself derived from a 28-item version [84]. It is used worldwide, and a recent systematic review found the WHO-5 to be highly clinimetrically valid, sensitive, and specific [85].

To assess the students’ self-esteem, the *Single-Item Self-Esteem Scale* (SISE) will be used [86], which has its origin in the Rosenberg Self-Esteem Scale [68] and has been shown to be a valid, reliable, economical, and practical instrument in German samples [56].

To evaluate participants’ ability to cope with internal or external stressors, we will assess their resilience, using the *Connor-Davidson Resilience Scale-short form* (CD-RISC 10), which is derived from the long version designed by Connor and Davidson in 2003 [87]. The CD-RISC is widely used and has good psychometric properties (*a* = .84) [57].

To assess protective factors for mental health in children and adolescents, we will use the German Scales for the Assessment of Protective factors (*Skalen zur Erfassung von Schutzfaktoren*) following the suggestions of Bettge and Ravens-Sieberer [55]. The scales have been validated in adolescents aged from 11 to 17 years and proved to be valid and practicable.

To measure non-suicidal self-injury (NSSI), we will use a shortened version of the nine-item *Deliberate Self-Harm Inventory* (DSHI-9), which was used in the SEYLE study. The original version of the DSHI [88] contains 17 items, and the DSHI-9 is an adaptation for adolescents [89]. To avoid confusing adolescents who do not harm themselves, we will introduce the questionnaire with a single question (“Have you ever hurt yourself with the intention of causing yourself physical pain?”). If they answer with “Yes”, NSSI will be explored further in terms of frequency, severity, and duration, using the DSHI-9. If they answer with “No”, NSSI will proceed to the next questionnaire. The internal consistency for the DSHI-9 lies between alpha = .66 and .85 [90].

In addition to demographic questions, the socioeconomic status will be captured using the *Family Affluence Sale* (FAS; [58] and the MacArthur Scale [91].

Finally, six questions regarding the COVID-19 pandemic will be asked, which explore stress factors, general mood, and well-being in families during the pandemic. These questions are from the Corona Health App, which was developed as part of a scientific cooperation between university partners, the Robert Koch Institute, and software companies (for detailed information, see www.corona-health.net).

### Plans to promote participant retention and complete follow-up {18b}

To promote retention throughout the study and maximise the completeness of data collection, we will give monthly feedback to the school heads, the involved teachers, and the participating students. Feedback may consist of a little insight into results of the first data collection, schedule reminders, a newsletter, or small business gifts (pens or stickers).

Should any student withdraw from the study for any reason, he or she must inform us, whether we are permitted to use the data already collected or we should delete it. If deletion is requested, the student must provide us with his/her pseudonym to enable deletion.

If non-retention occurs and more than one follow-up data assessment is missing for a student, this student will not be included in the analysis.

### Data management {19}

#### Data entry

Within the study *LessStress,* all data will be entered electronically. This will usually be accomplished using the participants’ own devices (cell phones, tablets, laptops) or they can use devices provided by their school. The data collection itself will take place online using the SoSciSurvey. This software solution is firmly established in academic research, e.g. at the University of Würzburg; the company headquarters are in Germany; and the software is in accordance with the European Union General Data Protection Regulations (GDPR—sometimes referred to as DSGVO in Germany). The data are directly recorded digitally and do not have to be entered separately by hand, which minimises the risk of input errors.

#### Coding process

Since there will be four measurement time points, the data cannot be collected completely anonymously, but are initially requested pseudo-anonymously. This enables the individual data records to be merged. The complete anonymisation of the data will take place after the 6-month follow-up. A written assignment of code to individual students is completely dispensed with in order to protect data. The data will not be passed on to third parties and the results will not be transmitted by email or post. The ethical and legal aspects according to the declaration of the World Medical Association in the current version will always be observed during data collection and processing.

#### Data security

The data will be transmitted via a secure network of the University of Würzburg. It will be impossible to assign the devices used to the individual adolescents by storing the IP address. The transmission of the data or the recording and storage of the results of the measurements are subject to a tested data protection concept. The digitally collected data will be encrypted at all times. Only authorised members of the study team will have access to the processes of data collection and monitoring of data processing.

#### Data storage

The anonymised data will be electronically stored on the servers of the computer centre of the University of Würzburg, password-protected, and deleted 4 years after the end of the study in accordance with data protection regulations. If a participant revokes his/her declaration of consent to participate in the study, the data obtained up to that point will be excluded from further analysis and destroyed, if they are still available in pseudonymised form. However, if the data have already been anonymised, they can no longer be assigned to the students. Accordingly, the data cannot be deleted. The adolescents and their legal guardians will be provided with detailed information in this regard.

### Confidentiality {27}

All information related to the study and its participants will be stored securely in either locked files or cabinets. Furthermore, information that contains personal identifiers (e.g. names) will be stored separately. Concerning this trial, there will be no list of names assigned to pseudonyms.

### Plans for collection, laboratory evaluation, and storage of biological specimens for genetic or molecular analysis in this trial/future use {33}

Not applicable. There are no plans for the collection of biological specimens and there will not be any genetic or molecular analysis.

## Statistical methods

### Statistical methods for primary and secondary outcomes {20a}

The intervention group will be compared to the waiting list control group in terms of the difference in change. The data structure will be clustered at different levels (school type, school, grade, class, student), and we must take this into account. To check for the impact of levels, the ICC will be calculated. If the ICC is bigger than 0.1, we will analyse the data using (general) multilevel models. If not, mixed ANOVAS will be performed. First, a null model will be built consisting of the dependent variable and the random intercept. The model fit will be given using the Akaike information criterion AIC. Based on the null model, the independent variables of interest will be included, for the primary outcome self-perceived stress (measured with the PSS-10) and for the secondary outcomes the remaining measure (DERS-SF, SCS, ISI, PHQ-4, CD-RISC, SEED, DSHI-6, SISE, WHO-5, protective factors, FAS III). The AIC will be used to compare the models to one another.

### Interim analyses {21b}

Interim analyses are planned for baseline data collection. Epidemiological studies will be reported and, if appropriate, a comparison with data from another region will be explored. The study will automatically end with the last data collection in the last class after 6 months. Access to these interim results and the final decision on the termination of the study will be at the discretion of the study management (MK, AB, CS).

### Methods for additional analyses (e.g. subgroup analyses) {20b}

We plan to split all of the participant data into subgroups for comparisons between males and females, different school types, age categories (younger vs. older determined by school grade), and healthy vs. clinically relevant. Depending on the variables, different methods will be used (*t*-test, Wilcoxon test, chi-squared), but we prefer multilevel modelling to explore associations between intervention effects and student characteristics.

### Methods in analysis to handle protocol non-adherence and any statistical methods to handle missing data {20c}

If parts of the protocol need to be changed, this will always occur in consultation with the study management and the cooperation partners. The final decision on any changes will be made by the study management. Missing data should be minimised by the online survey. However, if this should occur, the procedure of multiple imputation will be chosen.

### Plans to give access to the full protocol, participant-level data, and statistical code {31c}

The datasets analysed during the current study and statistical code are available from the corresponding author on reasonable request, as is the full protocol (note: this is only possible if the school board in charge approves this).

## Oversight and monitoring

### Composition of the coordinating centre and trial steering committee {5d}

*Principal investigator and research physician*: Christin Scheiner, Arne Bürger, Andrea Daunke, and Marcel Romanos—design and conduct of LessStress, preparation of protocol and revisions, organising steering committee meetings, publication of study reports, and members of TMC

*Steering committee (SC)*: Michael Kölch, Arne Bürger, and Andrea Daunke—agreement of final protocol, all lead investigators will be steering committee members, recruitment of participants and liaising with the principal investigator [Michael Kölch], reviewing the progress of the study, and if necessary agreeing changes to the protocol

*Trial management committee (TMC)*: Michael Kölch, Christin Scheiner, and Arne Bürger—study planning, organising steering committee meetings, advice for lead investigators, data verification, and budget administration

*Data manager*: Christin Scheiner, Andrea Daunke, and Marcel Romanos—maintenance of trial IT system and data entry and data verification

*Lead investigators*: Arne Bürger and Michael Kölch

### Composition of the data monitoring committee, its role, and reporting structure {21a}

As the trial will not be conducted in patients or any at-risk group, and as its duration is relatively short (time from the first to last data assessment is 6 months), there will not be a data monitoring committee (DMC). In addition, the trial cannot be modified once the intervention group has started. Of course, after each data assessment, we will check whether everything has ensued satisfactorily. There are no competing interests and the data are independent from the sponsor.

### Adverse event reporting and harms {22}

We do not expect any harms caused by the questionnaires or the prevention programme itself. However, we do offer consulting for students who are in need of a higher level of psychiatric care. The Department of Child and Adolescent Psychiatry, Neurology, Psychosomatics and Psychotherapy, University Medical Centre Rostock, can offer appointments locally and the University Hospital of Wuerzburg, Center of Mental Health, Department of Child and Adolescent Psychiatry, Psychosomatics and Psychotherapy, can offer consulting appointments online. Furthermore, the webpage and the brochure every student receives contain numbers of hotlines, where help is available 24/7. If any adverse events occur during the time of the trial, we will collect and document them and report the type of event as well as the managing process. Furthermore, during the training, teachers will be instructed about dysfunctional coping behaviour and how to deal with this in terms of the dialectical behavioural therapy for adolescents (DBT-A).

### Frequency and plans for auditing trial conduct {23}

There will be team meetings on a monthly basis within the members of the working group. Audits with the cooperation partners and with the sponsor will take place if necessary, but at the latest after the last follow-up at 6 months.

### Plans for communicating important protocol amendments to relevant parties (e.g. trial participants, ethical committees) {25}

Any modifications to the protocol will require a formal amendment to the ethics committee in Rostock as well as the Ministry of Education of Rostock.

## Dissemination plans {31a}

We plan to report all results, no matter the outcome following the guidelines of good scientific practice. Furthermore, we will endeavour to give easy access to the prevention programme *LessStress* for use in other schools.

## Discussion

According to the latest figures, over 20 million people in Germany are affected by mental illness (prevalence of 27.8%), including 2.7 million children and adolescents [92]. The goal of universal prevention is to increase competencies within a large population without prior selection. We want to contribute to this through the prevention programme *LessStress*. The advantages of *LessStress* lie in its short implementation time, the integration into normal school lessons, and the easy training of already existing school staff. If effects can be proven, a step would be taken towards keeping children and adolescents healthy in the school setting. If *LessStress* does not prove to be effective, it is important to find out what we can improve to bring scientifically evaluated and effective prevention programmes into schools. At the same time, some of the benefits of *LessStress* should also be seen as limitations. In particular, the short implementation time and the implementation by teachers may influence our outcomes. Moreover, the different schools with different requirements for the students could have a negative impact on this study. However, with our adjusted case number estimation, even small effects will be visible, insofar as they exist.

## Trial status

Protocol version 1, 04 November 2021

Start recruitment: 1 October 2022

Approximate date when recruitment will be completed: July 2023

## Data Availability

All principal investigators will be given access to the cleaned data sets. As this is a multicentre study, only the steering group has access to the full trial dataset. The data will remain on the server of the Data Centre of the University of Wuerzburg, where it will be safe, crypted, and not available for third parties. Any data required to support the protocol can be supplied on request, if the School Board in charge approved this.

## Abbreviations

CD-RISC 10: Connor-Davidson Resilience Scale
DERS-SF: Difficulties in Emotion Regulation Short Form
DMC: Data Monitoring Committee
DSHI-9: Deliberate Self-Harm Inventory 9-item version
FAS III: Family Affluence Scale
GAD-2: Generalized Anxiety Disorder Screening with 7 items
GDPR: European Union’s General Data Protection Regulations
HrQoL: Health-related quality of life
ICC: Intraclass correlation coefficient
IG: Intervention group
ISI: Insomnia Severity Index
KKH: Kaufmännische Krankenkasse
MBSR: Mindfulness-based stress reduction
PHQ: Patient Health Questionnaire
PSS-10: Perceived Stress Scale 10-item version
QoL: Quality of life
RCT: Randomised controlled trial
SCS: Self-Compassion Scale
SEED: Short Evaluation of Eating Disorders
SISE: Single-Item Self-Esteem
SFF: Schutzfaktoren Fragebogen (protective factors questionnaire)
WHO-5: World Health Organization 5 items for Well-Being
WL: Waiting list
